# Testing Affordable Strategies for the Computational Study of Reactivity in Cysteine Proteases: The Case of SARS-CoV-2 3CL Protease Inhibition

**DOI:** 10.1021/acs.jctc.2c00294

**Published:** 2022-05-13

**Authors:** Carlos A. Ramos-Guzmán, José Luis Velázquez-Libera, J. Javier Ruiz-Pernía, Iñaki Tuñón

**Affiliations:** † Departamento de Química Física, Universitat de Valencia, Burjassot, Valencia 46100, Spain; ‡ Departamento de Bioinformática, Facultad de Ingeniería, Centro de Bioinformática, Simulación y Modelado (CBSM), Universidad de Talca, Talca 3460000, Chile

## Abstract

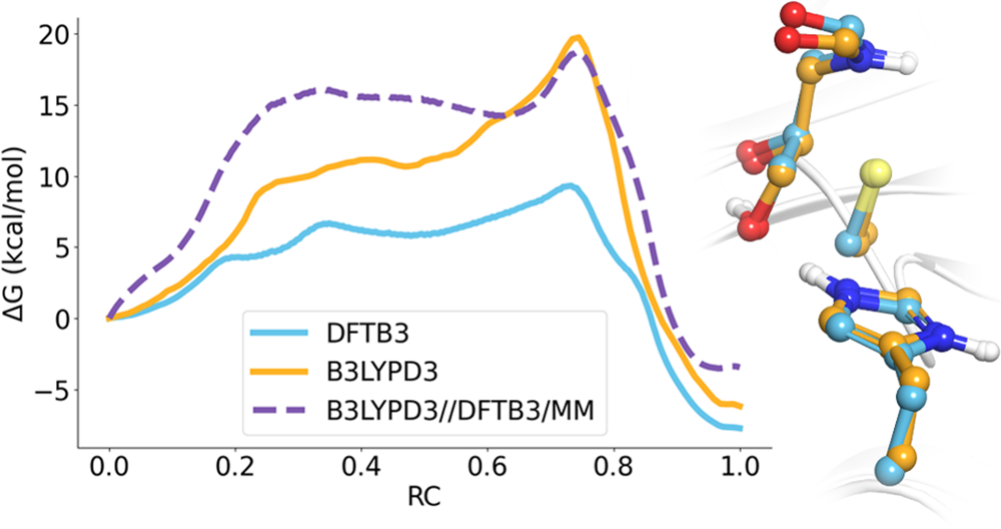

Cysteine proteases are an important target for the development of inhibitors that could be used as drugs to regulate the activity of these kinds of enzymes involved in many diseases, including COVID-19. For this reason, it is important to have methodological tools that allow a detailed study of their activity and inhibition, combining computational efficiency and accuracy. We here explore the performance of different quantum mechanics/molecular mechanics methods to explore the inhibition reaction mechanism of the SARS-CoV-2 3CL protease with a hydroxymethyl ketone derivative. We selected two density functional theory (DFT) functionals (B3LYP and M06-2X), two semiempirical Hamiltonians (AM1d and PM6), and two tight-binding DFT methods (DFTB3 and GFN2-xTB) to explore the free energy landscape associated with this reaction. We show that it is possible to obtain an accurate description combining molecular dynamics simulations performed using tight-binding DFT methods and single-point energy corrections at a higher QM description. The use of a computational strategy that provides reliable results at a reasonable computational cost could assist the in silico screening of possible candidates during the design of new drugs directed against cysteine proteases.

## 1 Introduction

Cysteine proteases represent one of the four main groups of peptide bond hydrolases. These enzymes are characterized by the use of a cysteine side chain as the nucleophile in charge of the proteolysis reaction. Cysteine proteases are found in all forms of life, including viruses, bacteria, protozoa, fungi, plants, and animals.^1,2^ In human cells, cysteine proteases mediate a wide variety of processes, from the bulk digestion of proteins to bone resorption and apoptosis.^3,4^ These enzymes are also present in many infectious agents, such as viruses, bacteria, and parasites, making them an attractive target for the development of cysteine protease inhibitors to be used as drugs.

**Figure 1:**
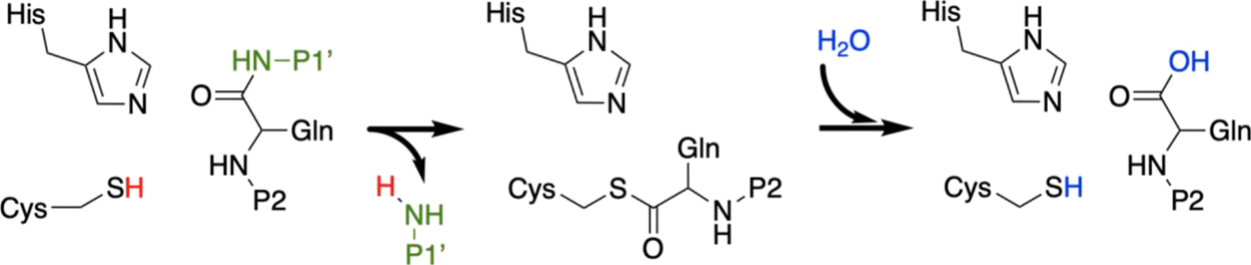
Schematic representation of the reaction mechanism in cysteine proteases showing the acylation and deacylation steps.

The reaction mechanism of these enzymes involves two basic steps: acylation and deacylation (see Figure 1).^5^ Their active sites contain a catalytic dyad formed by a cysteine and a histidine. While the former performs the nucleophilic attack, the latter can act as a proton acceptor/donor during the reaction mechanism. The nucleophile is activated after deprotonation that leads to the formation of the thiolate anion. This anion attacks the electrophilic carbon of the reactive bond, while the leaving amino group is protonated, resulting in the cleavage of the peptide bond. This leads to the release of the first reaction product and the formation of a thioester intermediate. The hydrolysis of this complex produces the second reaction product and the recovery of the initial state of the enzyme. Different variants of this basic reaction mechanism have been described for different enzymes.^6−8^ Main differences are associated with the protonation state of the catalytic dyad (both residues being neutral or forming an ion pair), the participation of the catalytic histidine in the proton transfer from the cysteine to the substrate and the concerted or stepwise nature of the basic steps.

**Figure 2:**
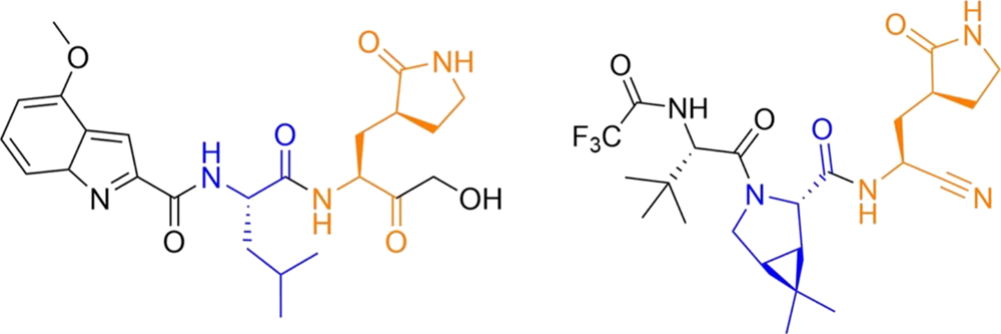
PF-00835231, left, and PF-07321332, right. P2 is highlighted in blue and P1 in orange.

The interest in the study of the reactivity and inhibition of cysteine proteases has increased since the COVID outbreak caused by the SARS-CoV-2 virus. This virus uses the transcription machinery of infected cells to translate its genomic material in two large polyproteins that must be cleaved to produce functional proteins for the new generation of viruses. This cleavage process is carried out by two cysteine proteases: the main or 3CL protease, in charge of cleaving the polyprotein in 11 of a total of 14 positions, and the papain-like protease (PPL).^9^ The 3CL protease is an interesting target for the development of inhibitors to be used as antivirals because this enzyme uses a recognition sequence not employed in any of the known human proteases (it cleaves the peptide bond after a Gln residue) and because it is well-conserved among different variants of the virus (the Omicron 3CL protease contains only one mutation, far from the active site, with respect to the wild type presenting similar catalytic properties).^10^ Pfizer already announced the development of two inhibitors of the SARS-CoV-2 3CL protease: PF-00835231, a hydroxymethyl ketone derivative,^11^ and PF-07321332 (or Nirmatrelvir), a nitrile-derived compound present in Paxlovid (see Figure 2).^12^ These compounds are peptidomimetics containing a warhead that is able to react with the nucleophilic cysteine, forming a stable acylenzyme complex with the 3CL protease of SARS-CoV-2. Both inhibitors present a γ-lactam ring at the P1 position and a hydrophobic group at P2, mimicking the natural substrate of the enzyme.

In general, cysteine protease inhibitors reduce the quantity of free enzymes, which can help to regulate some cellular processes or to interrupt the replication cycle of infectious agents. Atomistic details of reactions in cysteine protease reactivity can provide valuable information for the development of efficient and specific covalent inhibitors for them. This knowledge can be obtained from a combination of experimental techniques (such as kinetic and structural studies) and computational approaches. Proper simulations of enzymatic reactions must be carried out, including the interactions of the reactive system with a dynamical environment formed by the protein and the solvent. These simulations are nowadays affordable using hybrid quantum mechanics/molecular mechanics (QM/MM) approaches, where a small part of the system is described using QM methods, while the rest is treated using MM.^13,14^ The bottleneck in the use of these methodologies is the need to repeat a QM calculation at each step of the simulation, which typically restricts current applications to low-cost QM methods, such as wave-function-based semiempirical Hamiltonians or density functional theory (DFT) tight-binding descriptions. However, the reaction mechanism of cysteine proteases (see Figure 1) involves the deprotonation of a sulfur atom and the nucleophilic attack of the corresponding anion on a carbonyl group, which can compromise the performance of low-cost QM approaches. Very recently, we explored the reaction mechanism of the natural substrate and several inhibitors with the 3CL protease of SARS-CoV-2 using higher-level DFT treatments based on the B3LYP and M06-2X functionals, with the 6-31+G* basis set and including D3 dispersion corrections.^8,15−18^ These simulations provided results in excellent agreement with experimental estimations but at a considerable computational cost, which limits the amount of configurations that can be sampled and/or the size of the QM region.

In this work, we have used our previous experience to explore the performance of different low-cost QM descriptions, based on semiempirical and tight-binding treatments, for the description of SARS-CoV-2 3CL protease reactivity. We will show that the combination of an adequate low-level treatment and single-point energy corrections at higher levels can provide results in very good agreement with more expensive DFT/MM simulations. These results pave the way for trustable simulations of the reactivity of cysteine proteases within affordable computational costs, a desirable goal for the incorporation of QM/MM simulations in future studies for the design of new and more efficient inhibitors of cysteine proteases, including those of SARS-CoV-2. In principle, similar strategies for the optimization of QM/MM protocols can be envisaged for the study of different enzymes. These approaches should be particularly interesting to evaluate the impact of modifications in the design of inhibitors against relevant pharmacological targets, as in the case of the SARS-CoV-2. Note that related strategies have been already successfully used to develop fast computational QM/MM assays for the study of class A β-lactamases^19^ and fatty acid amide hydrolase.^20^

## 2 Methods

In order to analyze the performance of different QM/MM schemes in the analysis of 3CL protease inhibition with PF-00835231, we followed the same computational strategy reported in our previous study.^18^ The noncovalent enzyme–inhibitor (EI) complex was built using the PDB structure 6XHM.^11^ Maestro^21^ and PROPKA^22^ were used for the H-bond assignments and to determine the most likely protonation states of the titratable residues at pH 7.4. Regarding the parameters employed to describe the hydroxymethyl inhibitor, they were obtained by following the nonstandard residue parameterization procedure implemented in Amber using the Antechamber^23^ program from the AmberTools18^24^ package. The restrained electrostatic potential method^25^ using the HF/6-31G* level was used to define the atomic charges of the inhibitor, while the standard residues were described using the ff14SB forcefield.^26^ The necessary information required to carry out the simulations with the PF-00835231 inhibitor can be found in Supporting Information (frcmod and prepin files). The system was formed by the EI complex with enough Na^+^ ions to neutralize the electrostatic charge of the system and a water box, in which the solute’s closest atom to the boundaries of the box is at a distance of at least 12 Å. For that procedure, the program *tleap* from the AmberTools18 suite was chosen. The inhibitor was present in both monomers of the enzyme.

We first carried out a classical molecular dynamics (MD) simulation of the EI complex. The standard procedure employed to simulate the system started by a series of minimizations using 500 steps of the steepest descent algorithm, followed by steps of the gradient conjugate method. From this minimized structure, the system was heated using a linear ramp from 0 to 300 K. The first 60 K was raised during 10 ps running Sander in CPU, followed by a 100 ps heating ramp from 60 to 300 K using the Amber19 GPU version of pmemd.^27,28^ During this procedure, the heavy atoms of the backbone are restrained using a harmonic restraint with a force constant of 20 kcal mol^–1^ Å^–2^. Then, the system was equilibrated in the *NPT* ensemble (300 K and 1 bar) for 7.5 ns. The Berendsen barostat was used to control the pressure, while the Langevin thermostat was chosen to keep the temperature controlled. During this simulation time, the force constant is decreased every 1.25 from 15 to 0 kcal·mol^–1^ Å^–2^ (in 3 unit steps) until the system runs restraint-free during the last 1.25 ns of the equilibration. Then, the system was simulated for 1 μs in the *NVT* ensemble. The SHAKE^29^ algorithm was used to constraint the distances of the bonds between heavy atoms and hydrogens. The time step was 2 fs. The electrostatic short-range interactions were evaluated using a 10 Å cutoff, while the long-range electrostatic interaction was evaluated using the particle mesh Ewald.^30,31^

To study the reaction for the formation of the covalent EI complex, a QM/MM description was employed. In these calculations, the B3LYP^32,33^ and M062X^34^ functionals with a 6-31+G* basis set and D3 dispersion corrections^35^ were selected to describe the QM region at the DFT level.^36,37^ PM6^38^ Hamiltonian was selected among wave function semiempirical descriptions, while GFN2-xTB^39^ and DFTB3^40^ methods were used as DFT tight-binding methods. The Sander–Gaussian interface^41^ was employed to run the QM/MM simulations using a modified version of the AmberTools18 code^42,43^ with Gaussian16^44^ to perform the DFT calculations. The QM region included the side chain of the residues in the catalytic dyad (His41 and Cys145), backbone of residue P1, and the hydroxymethyl P1’ fragment of the inhibitor. The boundary between the QM/MM regions was described using the link atom approach. For all the QM–MM interactions, the cutoff radius used was 15 Å.

In the chemical reaction under study, several degrees of freedom change simultaneously from reactants to products. For this reason, we selected a methodology that help us to explore the associated multidimensional free energy surface (FES). In order to solve the curse of dimensionality, the adaptive string method (ASM)^45^ was chosen to find the minimum free energy path (MFEP). In this method, the MFEP can be traced on a multidimensional FES of arbitrary dimensionality defined by a selected set of collective variables (CVs) defined by the bond lengths of all the bonds being formed, broken, or whose formal order changes during the process, as it is the case of the carbonyl bond (see below). Once the MFEP is located, the path is used to define a Path Collective Variable (Path-CV, denoted as *s*), which is a function that increases monotonically, while the system moves along this path from reactants to products. This makes the Path-CV an adequate reaction coordinate for free energy calculations. As a single reaction coordinate is used, the computational cost is independent of the number of CVs describing the chemical events.

To locate the MFEP, a string that connects the regions of reactants and products is defined. In our case, the string is formed by a set of 96 replicas of the system (string nodes) evenly separated along the path. Every replica is a QM/MM restrained MD simulation. At every simulation step, each node is moved to a region of a lower energy according to their free energy gradient while keeping them equidistant along the string. This series of steps is repeated until the string converges to its MFEP displaying a RMSD below a 0.1 amu^1/2^ Å for at least 1 ps. Replica exchange attempts between nodes were made every 50 steps to increase the convergence. The converged path was then used to define the reaction coordinate (*s*) to trace the corresponding reaction free energy profile along the MFEP. For the system in which the QM region was described using a DFT level of theory, every node run for 10 ps and the sampled values were integrated with WHAM^46^ to obtain the free energy profile along the Path-CV. For the semiempirical calculations, this integration was made over longer simulation times of 80–100 ps. Details about simulation times and the computational cost of each method are provided in Table S1. The force constant values employed to bias the ASM simulations were obtained on-the-fly.^45^ This help us to obtain a homogeneous probability density distribution of the reaction coordinate. During the string simulations, the masses of the interchanging protons are set equal to 2 amu, and the time step employed was of 1 fs. Because the total length of the reaction coordinate *s* was different for each QM/MM method, we present the results as a function of a renormalized reaction coordinate (RC = (*s* – *s*_min_)/(*s*_max_ – *s*_min_)) that takes values between 0 and 1 in all the cases.

## 3 Results

### 3.1 QM/MM Descriptions

**Figure 3:**
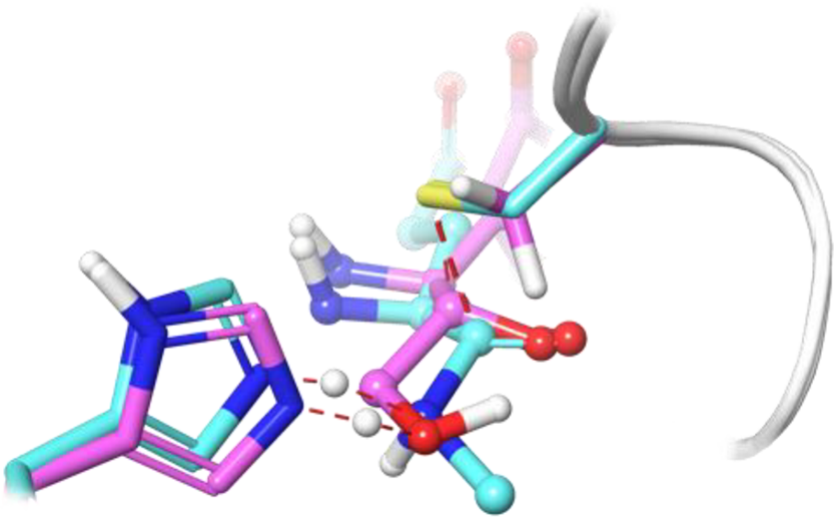
Comparison of the TS structures for the reaction of the 3CLpro with its natural substrate in cyan and the PF-00835231 in pink. Catalytic dyad in tube representation.^18^

The catalytic reaction in 3CL protease includes the nucleophilic attack of Cys145 to the electrophilic carbon atom of the substrate and a proton transfer from His41 to the substrate.^8,15−18^ The nucleophilic attack requires the activation of Cys145 by means of a proton abstraction carried out by the catalytic His41, resulting in the formation of an ion pair (IP: Cys141-S^–^ His41H^+^). When a peptide substrate is present, this proton is then transferred directly from His41 to the amino group of the target peptide bond.^8^ When an inhibitor is present, its protonation is mediated by a water molecule that occupies the position of the amino group in the natural substrate.^15−17^ In the case of PF-00835231, the hydroxyl moiety occupies the position of the amino group, and then the proton transfer from His41 to the substrate takes place through the hydroxyl group (see Figure 3).^18,47^

**Figure 4:**
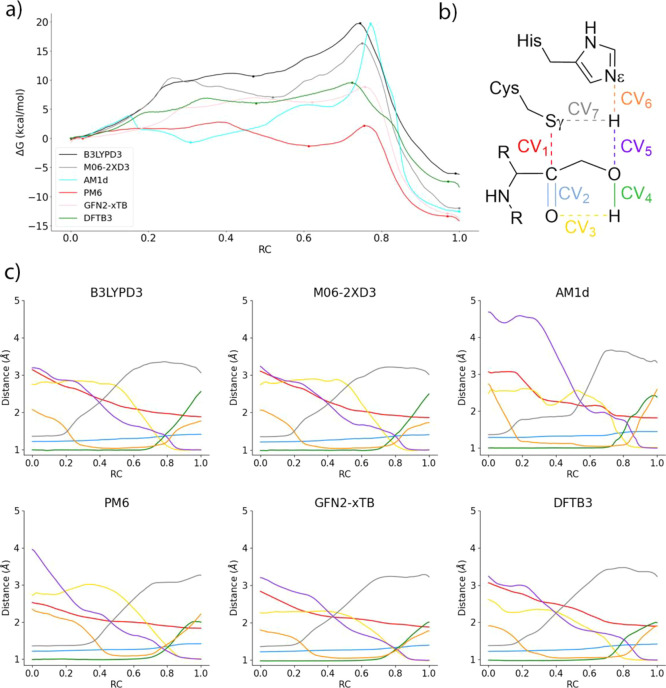
(a) Free energy profiles obtained with different QM levels for the inhibition of 3CLpro with PF-00835231; (b) representation of the CVs used to obtain the MFEPs; and (c) evolution of the CVs along the string nodes for the converged MFEPs.

The results obtained for the determination of the minimum free energy path (MFEP) for 3CL protease inhibition by PF-00835231 using different descriptions for the QM subsystem are presented in Figure 4. All of them agree in the timing of the key events during this reaction: the reaction is initiated with the proton abstraction from Cys145 by His41 (resulting in the IP formation), followed by the nucleophilic attack and then the proton transfer from His41 to the carbonyl oxygen atom of the substrate, using the hydroxyl group as a proton shuttle. However, different methods disagree in several details.

**Figure 5:**
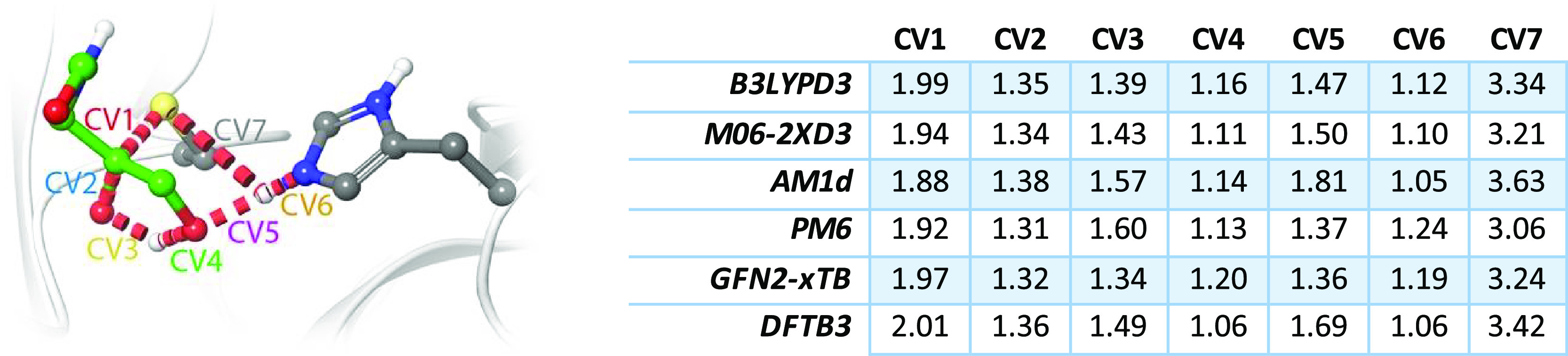
Representation of the rate-limiting TS for 3CLpro inhibition (left) and values (in Å) of all the CVs corresponding to the MFEP at the reaction TS, obtained with different QM approaches (right).

Both DFT and MM descriptions (using the B3LYP and M06-2X functionals, both with D3 corrections) provide an almost identical geometrical description of the reaction mechanism, as indicated by the evolution of the CVs along the MFEP (see Figure 4c). Proton transfer from Cys145 to His41 clearly precedes the nucleophilic attack, as reflected in the value of the Sγ–C distance, around 2.7 Å when the proton is at equal distances from the donor and acceptor. The nucleophilic attack is then completed reducing the Sγ–C distance below 2 Å, which is followed by the proton transfer from His41 to the inhibitor. Regarding the proton relay mechanism, the proton transfer from the hydroxyl group to the carbonyl oxygen atom slightly precedes the proton transfer from His41 to the hydroxyl group. The main difference between both DFT descriptions is found in the free energy profiles (see Figure 4a). The M06-2X functional predicts a more stable IP intermediate than B3LYP (7.0 vs 10.7 kcal mol^–1^, see Table 1). In fact, at the B3LYP level, the IP appears as a flat free energy region (see Figure 4a, black line), while at the M06-2X level, the IP is a clear free energy minimum (see Figure 4a, gray line). M06-2X also results in a smaller activation free energy (16.3 vs 19.7 kcal mol^–1^, see Table 1) and a more exergonic process (−12.0 vs −6.2 kcal mol^–1^). The reaction transition state (TS) at the B3LYP level and the rate-limiting TS at the M06-2X level appear with very similar values of the CVs (see Figure 5). PDB files with all the TS structures are provided as Supporting Information. At the TS, the Sγ–C bond (Sγ–C) is almost completely formed, displaying a bond length of 1.99/1.94 Å, at the B3LYP/M06-2X level. The two proton transfers, from His41 to the hydroxyl group and from this to the carbonyl oxygen atom, are not very advanced at the TS. The His41Nε–H distance has been lengthened up to 1.12/1.10 Å, while the distance of this proton to the hydroxyl oxygen atom of the inhibitor is 1.47/1.50 Å. Regarding the proton transfer from the hydroxyl group to the carbonyl oxygen, the distances of the proton to the donor and acceptor atoms of 1.16/1.11 and 1.39/1.43 Å, respectively, are just slightly more advanced than the transfer from His41.

**Table 1:** Free Energies (in kcal·mol^–1^) for Different Species Appearing along the Reaction Path Relative to Reactants (R) Using B3LYPD3, M06-2XD3, GFN2-xTB, DFTB3, AM1d, and PM6 Levels for the QM Region

	R	IP	TS	P
B3LYPD3	0.0	10.7	19.8	–6.2
M06-2XD3	0.0	7.0	16.3	–12.0
AM1d	0.0	–0.7	19.7	–12.5
PM6	0.0	–1.3	2.2	–13.4
GFN2-xTB	0.0	6.2	8.8	–12.9
DFTB3	0.0	6.0	9.6	–7.4

Although the free energy profiles are not dramatically different, it is of interest to discuss the reliability of both functionals to characterize the energetics of the reaction under study. One must consider that we are interested in a multistep process and that it can be difficult to find a functional describing equally well all the steps. The B3LYP functional is one of the best functionals to characterize the energetic of the proton transfer between cysteine and histidine when compared to higher-level electronic structure methods.^48^ On the other hand, it is also known that this functional can present problems to describe correctly enolate and carbanion intermediates.^49,50^ However, this limitation is not much important in the system studied because the nucleophilic attack takes place together with the proton transfer from His41 to the substrate. In this case, an enolate is not fully formed since the negative charge is compensated by the incoming proton. In fact, the Sγ–C distance presents an almost identical evolution along the MFEP with the two functionals presented here (see Figure 4c). Our previous results with other inhibitors also indicate that B3LYP correctly describes the nucleophilic attack of a thiolate to a Michael acceptor if this is accompanied by a proton transfer.^15^ On the experimental side, there are no direct measurements of the rate constant for the inhibition of 3CL protease with PF-00835231, and then, the experimentally derived activation free energy is not known. However, the free energy barrier derived from the experimental rate constant for a closely related aldehyde inhibitor, GC373, at 30° is 21.1 kcal·mol^–1^,^18,51^ a value close to our B3LYP estimation for PF-00835231. The agreement also obtained between the theoretical and experimental values of the activation free energy for the reaction of the natural substrate^8^ and the N3 inhibitor^15^ with 3CL protease provides further support to the validity of the B3LYP functional in the study of these processes.

We also located the MFEPs corresponding to two density functional tight-binding methods in combination with the MM potential (GFN2-xTB/MM and DFTB3/MM). The geometrical descriptions obtained from these two methods agree well with the DFT/MM results previously obtained. The main difference with respect to the DFT/MM results, which does not affect the description of the chemical steps, is the formation of a hydrogen bond between the hydroxyl group and the carbonyl oxygen atom of the substrate at the reactant state of the process at the GFN2-xTB level (note the smaller value of CV3 in Figure 4c for the reactant state when using GFN2-xTB). The evolution of the bond-breaking and bond-forming distances follows the same timing than that observed when B3LYP and M06-2X functionals are employed. The reaction is triggered by the abstraction of the proton of Cys165 by His41, followed by the formation of the Sγ–C bond and the protonation of the substrate mediated by the hydroxyl group. GFN2-xTB shows a shorter Sγ–C distance when the proton is being transferred to His41, about 2.4 Å, while DFT and DFTB3 calculations predict a Sγ–C distance of about 2.7 Å. GFN2-xTB also predicts a slightly more concerted character for the proton transfers between His41, the hydroxyl group, and the carbonyl oxygen atom of the substrate, as observed in the evolution of the respective CVs (CV3, CV4, CV5, and CV6) in Figure 4c. The shape of the free energy profiles obtained at GFN2-xTB and DFTB3 levels (see Figure 4a) also agrees qualitatively with the DFT results, showing a TS corresponding to the proton transfer from His41 to the substrate, although the barriers are substantially smaller (8.8 and 9.6 kcal·mol^–1^, using GFN2-xTB and DFTB3, respectively, see Table 1). Both tight-binding methods also agree with B3LYP results in describing the IP as a metastable species presenting a flat free energy landscape around (see pink and green lines in Figure 4a).

The two semiempirical methods used in this work (AM1d and PM6) also agree in the general description of the reaction mechanism for 3CL protease inhibition with PF-00835231. However, the MFEPs located using these QM descriptions present noticeable differences with respect to the DFT/MM results. At the PM6 level, the Sγ–C distance at the reactant state is substantially shorter than that with other methods, about 2.5 Å; while using other QM levels, the distance is closer or longer than 3.0 Å (see red lines in Figure 4c). Using PM6, when the proton of the Cys145 side chain is being abstracted by His41, Sγ–C is almost completely formed; while in other cases, the bond is formed only after the deprotonation of the Sγ atom. Regarding the proton transfer from His41 to the substrate, the PM6 method predicts a reversed ordering for the proton relay mechanism: the proton transfer from His41 to the hydroxyl group precedes the proton transfer from this group to the carbonyl oxygen atom of the substrate (see the evolution of CV3, CV4, CV5, and CV6 in Figure 4c). Instead, the AM1d Hamiltonian predicts the same ordering than the DFT/MM results. However, this last method fails to describe the formation of the Sγ–C bond. First, the proton transfer from Cys145 to His41 is decoupled from the nucleophilic attack; the Sγ–C distance starts to decrease only once the sulfur atom has been deprotonated (see red line in Figure 4c). Second, the Sγ–C bond is only fully formed once the carbonyl oxygen atom has been protonated, reflecting the inaccurate description of the carbanionic species with this semiempirical Hamiltonian. The energetic description is not much better. With both semiempirical Hamiltonians, the IP form is predicted to be a minimum even more stable than the reactants, with relative free energies of −1.3 and −0.7 kcal·mol^–1^ (see Table 1) with respect to the reactants, at the PM6 and AM1d levels, respectively. Instead, the AM1d Hamiltonian predicts a much more reasonable activation free energy. The free energy differences of the rate-limiting TSs with respect to the reactants are 2.2 and 19.7 kcal·mol^–1^. In all cases, the reaction is clearly exergonic.

### 3.2 Dual-Level Approach

According to our previous results, the DFTB3/MM and GFN2-xTB/MM levels provide a geometrical description of the 3CL protease inhibition process close to that obtained at higher, but computationally much more expensive, levels. In geometrical terms, the MFEPs obtained using DFTB3 and GFN2-xTB are close to those obtained using B3LYP and M06-2X functionals, while they can be obtained at a fraction of their computational cost. However, as discussed above, these tight-binding methods clearly underestimate the activation free energy and the free energy cost required to form the ionic pair form of the catalytic dyad.

The previous observation suggests a computational scheme to treat the inhibition of 3CL protease, and hopefully other chemical reactions in cysteine proteases, based on the combination of tight-binding/MM simulations with a posteriori single-point energy corrections at a higher DFT or ab initio level. This combination requires much less computational effort than direct DFT/MM or ab initio/MM schemes, allowing us to explore longer simulation times and/or larger QM regions if required. In this dual-level treatment, the free energy profile obtained at the DFTB3/MM or GFN2-xTB/MM levels is corrected according to the following scheme:
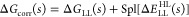
1where *s* is the Path-CV used to obtain the free energy profile along the MFEP, Spl denotes a spline function, and Δ*E*_LL_^HL^(*s*) is a correction term taken as the difference between a high-level (HL) energy calculation for the QM subsystem at the coordinate *s* and the low-level (LL) result. In our case, we corrected the DFTB3/MM and GFN2-xTB/MM values (our LL methods) by means of single-point energy calculations at the B3LYPD3/MM level (the HL method). The structures selected to calculate the correction were obtained after LL optimizations at different *s* values corresponding to the final positions of the string nodes. The optimizations along the Path-CV were done iteratively following forward and backward directions until the convergence of consecutive energy profiles. For each node of the string, we carried optimizations combining the steepest descent and the conjugated gradient optimization algorithms, as implemented in Amber. In these optimizations, harmonic restraints are applied along the Path-CV and orthogonally, relaxing both the QM and MM regions. We refer to these correction schemes as HL//LL/MM.

**Figure 6:**
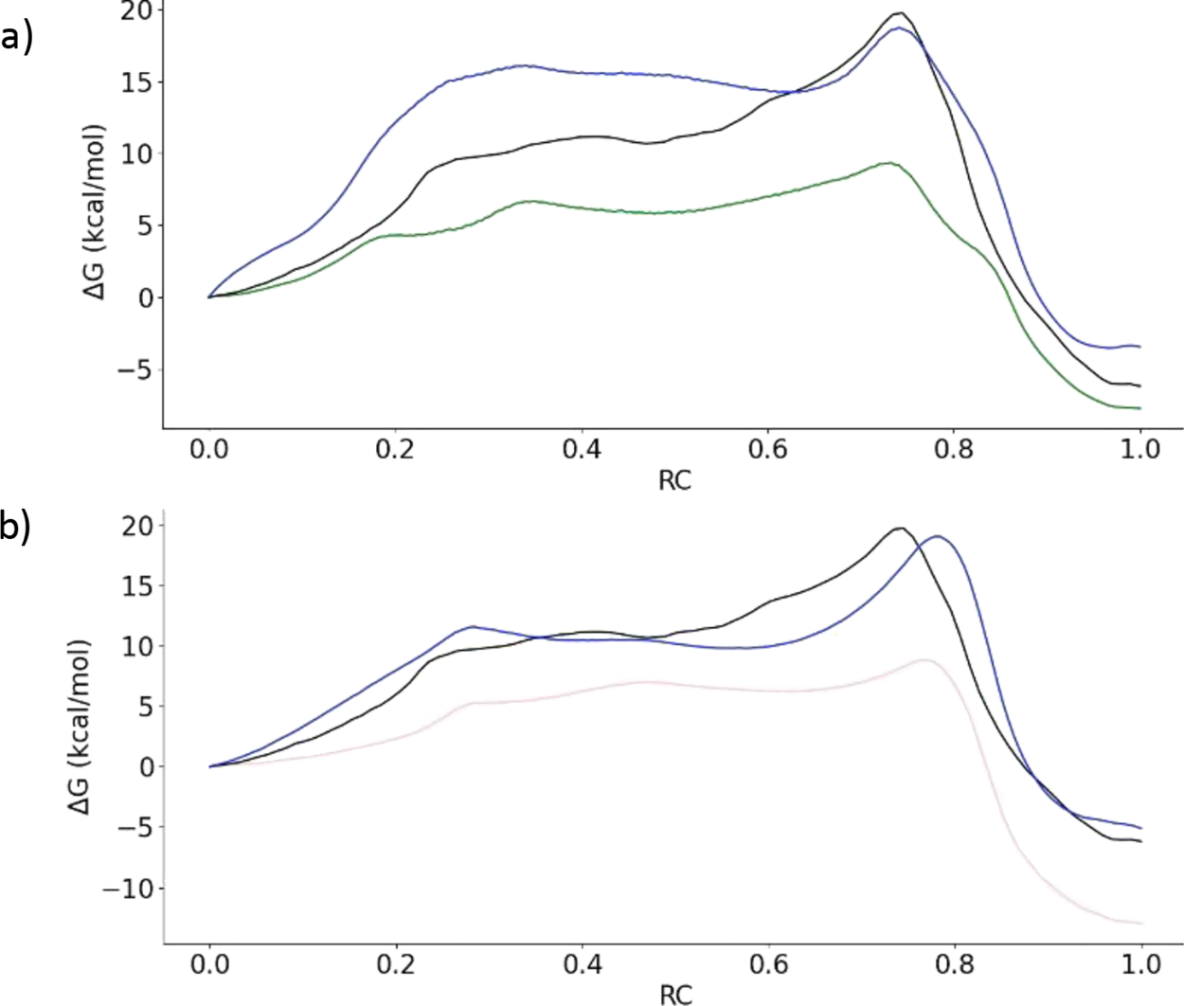
Free energy profiles for 3CLpro inhibition with PF-00835231 obtained with different QM/MM methods: (a) B3LYPD3/MM (in black), DFTB3/MM (in green), and the corrected B3LYPD3//DFTB3/MM profile using eq 1 (in blue). (b) B3LYPD3/MM (in black), GFN2-xTB/MM (in pink), and the corrected B3LYPD3//GFN2-xTB/MM profile using eq 1 (in blue).

The resulting free energy profiles are presented in Figure 6. The corrected B3LYPD3//DFTB3/MM free energy profile correctly reproduces the free energy height of the rate-limiting TS at the B3LYPD3/MM level, predicting an activation free energy of 18.7 kcal·mol^–1^, to be compared with the reference value of 19.7 kcal·mol^–1^. The main difference with respect to the B3LYPD3/MM free energy profile is the overestimation of the free energy associated with the IP, probably due to an underestimation of the magnitude of the QM–MM interactions stabilizing the charge separation at the lower level. Instead, the corrected GFN2-xTB/MM free energy profile reproduces better the relative free energy of the IP when compared to the B3LYPD3/MM profile and also the activation free energy (19.1 vs 19.7 kcal·mol^–1^). Regarding the geometry of the TS, the MFEP values reported in Table 2 show that this simple correction scheme does not necessarily result in a TS geometry closer to the target (the one obtained using the B3LYP functional), although the initial geometries provided by the DFTB3 and GFN2-xTB methods were already quite close and then are difficult to improve. The formation of the S–C bond is almost equally well described in all the methods presented in Table 2. Regarding the hydroxyl-mediated proton transfer from His41 to the substrate, the correction scheme provides an improved value over the DFTB3 description, while it is slightly too advanced when corrections are applied to the GFN2-xTB MFEP.

**Table 2:** Values (in Å) of All the CVs Corresponding to the MFEP at the TSs Obtained with the Different QM Levels, Including B3LYPD3, Tight-Binding Methods (DFTB3 and GFN2-xTB), and Correction Schemes (B3LYPD3//DFTB3 and B3LYPD3//GFN2-xTB)

	CV1	CV2	CV3	CV4	CV5	CV6	CV7
B3LYPD3	1.99	1.35	1.39	1.16	1.47	1.12	3.34
DFTB3	2.01	1.36	1.49	1.06	1.69	1.06	3.42
B3LYPD3//DFTB3	1.97	1.37	1.25	1.20	1.64	1.08	3.44
GFN2-xTB	1.97	1.32	1.34	1.20	1.36	1.19	3.24
B3LYPD3//GFN2-xTB	1.96	1.32	1.30	1.23	1.33	1.21	3.24

According to these results, the B3LYPD3//DFTB3/MM and B3LYPD3//GFN2-xTB/MM combinations seem to be interesting low-cost strategies to study the reaction mechanism for 3CL protease inhibition, and other reactions taking place in cysteine proteases, provided that the energy results are corrected *a posteriori*. Although the activation free energy would be clearly underestimated, more accurate results can be readily obtained adding single-point energy corrections at higher QM levels, not necessarily restricted to DFT calculations. Note that due to the accuracy of the DFTB3 and GFN2-xTB methods to describe the geometry of the structures appearing along the MFEP, this correction could be limited to the reactant and TS if we were only interested in the activation free energy. This provides an efficient and agile strategy to test the impact of new inhibitors’ designs on the kinetics of the formation of the covalent complex, facilitating the in silico screening of putative drugs.

## 4 Conclusions

Cysteine proteases are an important group of enzymes in charge of proteolysis reactions using the side chain of a catalytic cysteine as the nucleophile forming a covalent bond with the electrophilic carbon atom of the target peptide bond. Because of the presence of sulfur and of intermediate charged species, the electronic description of the reactions taking place in the active sites of these enzymes may be difficult to describe correctly at a reasonable computational cost. This problem becomes even more critical considering that enough sampling of the protein environment must be considered to properly take into account all the rearrangements needed to accommodate the charge transfer processes taking place during these chemical reactios. That is, one simultaneously needs an accurate electronic description combined with long enough simulation times.

In this work, we have compared the performance of different QM/MM schemes in the study of the 3CL protease inhibition by PF-00835231, a hydroxy methyl ketone compound that forms a covalent bond in the enzymatic active site between the electrophilic carbon atom of the ketone group of the inhibitor and the Sγ atom of the catalytic cysteine. We employed the string method to locate the MFEP using different descriptions for the QM subsystem: two DFT functionals with D3 corrections (B3LYP and M06-2X), two tight-binding methods (DFTB3 and GFN2-xTB), and two semiempirical Hamiltonians (AM1d and PM6). The analysis of the evolution of the CVs used to obtain the MFEP (the distances of all those bonds being broken, formed, or whose bond order changes during the reaction) demonstrates that the two DFT functionals provide a very similar geometrical description of the reaction, although some differences are found regarding the stability of the ion pair, the activation, and the reaction free energies. The use of the two tight-binding methods to describe the QM region results in a geometrical description of the reaction in very good agreement with the DFT results, in particular for the DFTB3 method. Instead, these methods produce activation free energies clearly underestimated with respect to the DFT values. Finally, the MFEPs obtained using the two semiempirical Hamiltonians present larger discrepancies with respect to the DFT results. These methods also overestimate the stability of the ionic pair form of the catalytic dyad.

The results presented here suggest that one can efficiently study the reactivity of the SARS-CoV-2 3CL protease, and probably other cysteine proteases, exploring the reaction mechanism using DFTB3/MM or GFN2-xTB/MM free energy calculations and then correct the activation free energies through single-point calculations using higher-level QM methods. Another possibility would be the use of reweighting schemes to introduce correction to the QM description using short, and then easily affordable, simulations at the reactants and TSs or to add energy corrections predicted by machine learning schemes. In any case, the possibility of having a methodology capable of providing reliable results at a reasonable computational cost paves the way for the use of QM/MM methods in the design of new drugs.

## Supporting Information Available

Convergence and sampling times for each MFEP and link to the files containing information for classical simulations and TS configurations (PDF)
