# Supplementary material for: Testing Affordable Strategies for the Computational Study of Reactivity in Cysteine Proteases: The Case of SARS-CoV-2 3CL Protease Inhibition
Source: J Chem Theory Comput. 2022 May 13;18(6):4005–13. doi: 10.1021/acs.jctc.2c00294 (PMC9115880; doi:10.1021/acs.jctc.2c00294)
Supplement: Supplementary file 1 — ct2c00294_si_001.pdf [file ct2c00294_si_001.pdf]

Supporting Information

Testing Affordable Strategies for the Computational Study of Reactivity in Cysteine Proteases. The Case of SARS-CoV-2 3CL Protease Inhibition

Carlos A. Ramos-Guzmán[a], José Luis Velázquez-Libera[a,b], J. Javier Ruiz-Pernía[a]*, Iñaki Tuñón[a]*

[a]Departamento de Química Física, Universitat de Valencia, 46100 Burjassot, Valencia, Spain.

[b]Departamento de Bioinformática, Facultad de Ingeniería, Centro de Bioinformática, Simulación y Modelado (CBSM), Universidad de Talca, Talca 3460000, Chile.

*To whom correspondence should be addressed:
ignacio.tunon@uv.es
j.javier.ruiz@uv.es

Table S1 S2
Information to download files S3

Table S1. Simulation times needed to converge the string and used to collect the sampling data for the reaction free energy profile and CPU times for each of the methods employed.

Method	Simulation time to converge the string[a] (in ps)	Simulation time accumulated for Free Energy Profile (in ps)	CPU time per step (in s)
B3LYPD3	10.5	10.0	232.2
M06-2XD3	3.0	10.0	345.1
AM1d	25.0	104.2	1.88
PM6	12.5	111.8	1.93
DFTB3	15.0	100.6	1.88
GFN2-xTB	29.2	81.0	2.01

[a]note that these times depends on the initial guess. For example, B3LYP simulations were started from DFTB3 results, while at the M06-2X level we employed the B3LYP results as initial guess.
bCPU times obtained for simulations running on a single CPU (Xenon Skylake processor)

All files are stored in the next link:
https://disco.uv.es/pub/efme/disco/SARS-CoV-2/3CLPRO_PF835231_TS/
and
https://covid.molssi.org//models/#transition-state-ts-structures-for-the-inhibitory-mechanism-of-the-pf-00835231-molecule-with-the-enzyme-3clpro-of-sars-cov-2

Name of the file	Description	
CL4_gaff.frcmod	Frcmod Amber file p2-p3 fragment	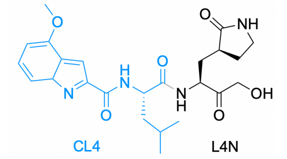
L4N_gaff.frcmod	Frcmod Amber file p1 fragment	
CL4.prepin	Prepin Amber file p2-p3 fragment	
L4N.prepin	Prepin Amber file p1 fragment	
B3LYP_TS.pdb	PDB file of the TS structure obtained at B3LYP level	
M062X_TS.pdb	PDB file of the TS structure obtained at M062X level	
AM1D_TS.pdb	PDB file of the TS structure obtained at AM1d level	
PM6_TS.pdb	PDB file of the TS structure obtained at PM6 level	
DFTB3_TS.pdb	PDB file of the TS structure obtained at DFTB3 level	
GFN2xTB_TS.pdb	PDB file of the TS structure obtained at GFN2-xTB level	
